# The Twin Forceps: A New Instrument for SILS

**DOI:** 10.1155/2015/361093

**Published:** 2015-08-04

**Authors:** Antonia Rizzuto, Mario Donnici, Paola Nudo, Basilio Sinopoli, Rosario Sacco, Guido Danieli

**Affiliations:** ^1^Università della Magna Græcia, Viale Europa, 86100 Germaneto, Italy; ^2^Calabrian High Tech (CHT) S.r.l., Via Ponte Bucci, Cubo 45C, 87036 Rende, Italy; ^3^Università della Calabria, Via Ponte Pietro Bucci, Cubo 45C, 87036 Rende, Italy

## Abstract

In the last ten years, the single incision laparoscopic surgery (SILS) is gaining more interest than the traditional laparoscopic surgery (LAP). Many studies make a comparison between the performances of the SILS and the LAP. The results show that the single incision laparoscopic surgery reduces pain, length of period of postoperative hospitalization, and loss of blood. This technique is also able to reduce the infection sites. In spite of many advantages, SILS reveals some problems: laparoscopic instruments triangulation and small workspace. The surgeon has to be more skillful to make a surgery in SILS because the surgeon has only three laparoscopic instruments and only one hole in the abdomen cavity. In this paper, a novel laparoscopic instrument to help the surgeon during a SILS operation is presented. This instrument is innovative forceps with double graspers. Different designs of this instrument are presented, with the final one which greatly simplifies both construction and operation. The initial experience in the laboratory with the innovative instrument is presented. The surgeon experienced in laparoscopic surgery and with the help of assistants performed a training program based on predetermined task performed in simulation box (laparoscopic box-trainer).

## 1. Introduction

In the past, the surgeon used to perform laparotomies, surgical procedure requiring a big incision in the abdominal wall to gain access into the abdominal cavity [1, 2].

Now, traditional laparoscopy is a less invasive surgical technique than laparotomy [3, 4]. In this method, the surgeon does not operate a surgery with the hands viewing directly the effect of his/her actions, but through instruments, observing the scene on a monitor. The surgeon makes a small incision about 1-2 cm long near to the navel (belly button). Then the abdomen is inflated with carbon dioxide gas. This makes it easier to see the internal organs with the laparoscope. To introduce other laparoscopic instruments, the surgeon makes one or more separate small incisions in the abdominal skin. These allow thin instruments to be introduced into the abdominal cavity. The laparoscopic instruments (graspers, scissor, and clip applier) have a diameter of 5–10 mm. So, in a traditional procedure (LAP), the surgeon can use two forceps to keep tissues in traction [5–11].

The evolution of surgery toward less invasive approaches brought about a new technique known by the acronym SILS (single incision laparoscopic surgery) [12, 13]. The surgeon makes a single incision in the navel (belly button) (about 20 mm) as the access port to the abdominal cavity. The surgeon then inserts trocars to allow the instruments insertion, but due to space limitation, only three instruments are currently used: a grasper, the laparoscope, and scissors or other surgical tools. In fact, even if it would be technically possible to use four rather than three instruments, the problem is not in the number of instruments, but in the coordination between the surgeons [14–16]. Four instruments require four hands, so that either a surgeon will hold the two graspers, and the second the optics and the surgical instrument, or the operator will hold a grasper and the surgical instrument, and the other the remaining instruments. In both cases the real problem is coordination between the two surgeons, beside the lack of space, having four hands close together while needing space to move the surgical instrument.

Unlike traditional laparoscopic surgery (LAP), in the SILS double tissues traction and a wide workspace are missed [17, 18].

**Figure 1:**
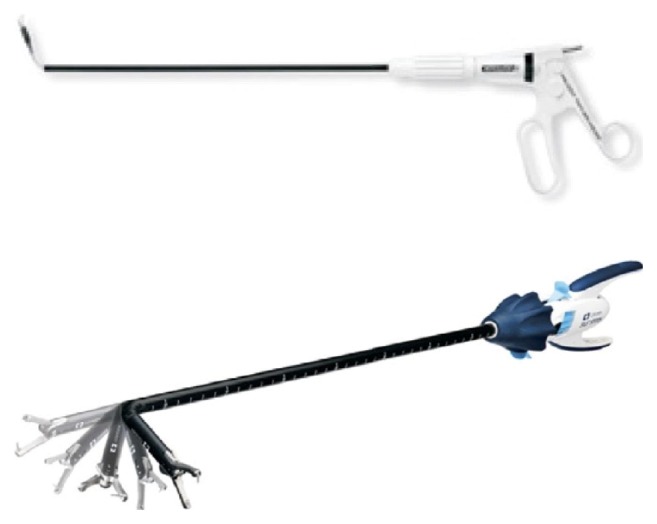
New laparoscopic forceps proposed for increasing manoeuvrability.

Due to the growing interest in this surgical technique, many manufacturers proposed innovative laparoscopic instruments, to improve triangulation, retraction, and patient safety. The first problem to resolve is the laparoscopic instrument triangulation. One of the early lines of an innovative product is the Covidien Roticulator [17, 19]. These instruments allow 0° to 80° articulation with 360° of axial rotation of the articulated angle. The handle articulates and can be locked, as shown in Figure 1.

All these laparoscopic instruments allow some triangulation but do not solve the problem of the double tissues traction.

**Figure 2:**
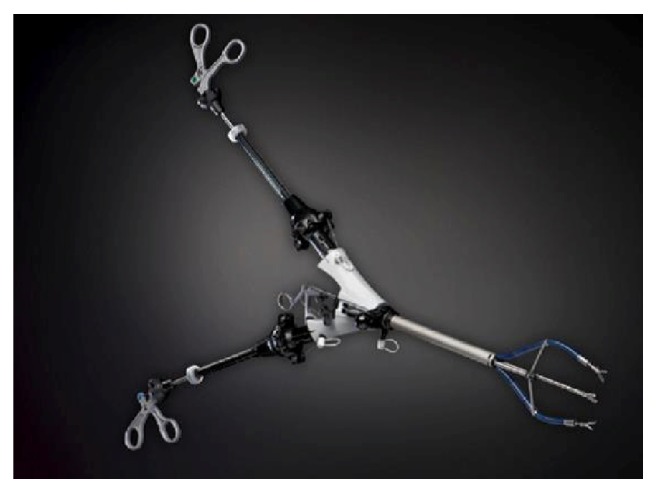
Spider surgical system.

Another example is Spider Surgical System: a single access device that allows using flexible laparoscopic instruments through flexible cannulas [17, 20, 21]. In particular, in the case of the Spider System, the surgical instrument is always kept in the middle between the two graspers, and this forces the grasper's position movement in order to change the surgical instrument position. Besides that, it is also noticeable that the Spider requires two hands to be actuated, or at least to move the hand from one side of the instrument to the other, losing control of the instrument during this process.  Figure 2 shows an image of Spider. This does not occur with our twin forceps, since surgical instrument and graspers are totally independent.

Therefore, in this paper, innovative double forceps, the need for which was felt by the surgeon, first author of the article, while the technical solution was found by the last author of the same, is illustrated.

## 2. The Initial Design

**Figure 3:**
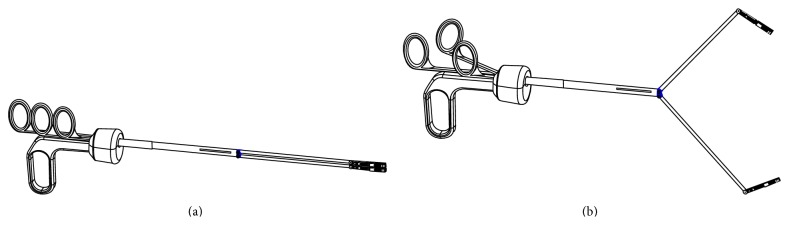
Laparoscopic twin forceps: (a) close and (b) open.

The forceps with double graspers is an innovation in surgical environment [22, 23]. According to the first idea of this instrument, its handle presented four rings, one lower and fixed and the other three on the upper part for controlling the instrument movements: (1) the central one is dedicated to the separation and rapprochement of the two arms, while the side ones are in charge of opening and closing the graspers (Figure 3). This instrument takes advantage of the traditional monotrocar to enter in the abdominal cavity. Once the forceps enters in the trocar (about 12 mm diameter), it is able to wide open the two arms, while the frames holding the forceps bend inward, so the surgeon can obtain the double tissues traction.

To obtain the tissues traction, a transmission system has been studied. The central ring is rigidly connected to a pulley, which controls, via transmission cables and a second pulley of smaller diameter, the arms opening. A manual rotation of 22.5 degrees of the central ring will correspond to a rotation of the arm of 45°. This allows the arms relative rotation with a maximum opening of 90°. Instead, the forceps counterrotation is produced by a couple of gears, whose rotation is also controlled by cable transmission commanded by the same pulley controlling the arms opening.

**Figure 4:**
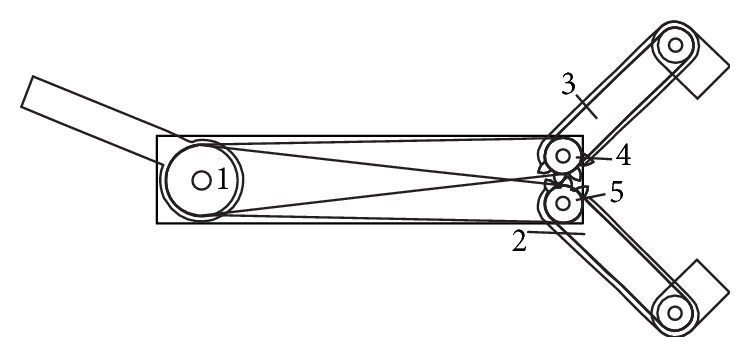
Scheme of the cables path.

As previously stated, the basic mechanism for opening the arms and counterrotating the forceps is composed of a mixed system of pulleys and gears, as shown in Figure 4, where the path of the cables is shown. In particular, two cables are wrapped around the drive pulley, fixed to the central command ring (1), the first transmitting motion to a lower pulley (2) part of the lower arm and presenting also a few teeth meshing with the second arm (3), and the second cable driving a second toothed pulley placed atop the forceps arms (4), which meshes with a second toothed pulley (5). Both pulleys on the right side have diameter half of that of the drive pulley. Since both arms are toothed, lifting the ring by a certain angle causes the arm pulley, due to the different diameter, to rotate in the same clockwise direction twice as much, while the upper arm, driven by the teeth, rotates by the same amount but in the opposite direction. Conversely, the upper pulley sitting on the top of the upper arm, also fixed to a gear, meshing with a second gear and pulley, rotates in the opposite direction. These gears are then connected through cables to the grasper holding frames, causing counterrotation of these with respect to the arms.

**Figure 5:**
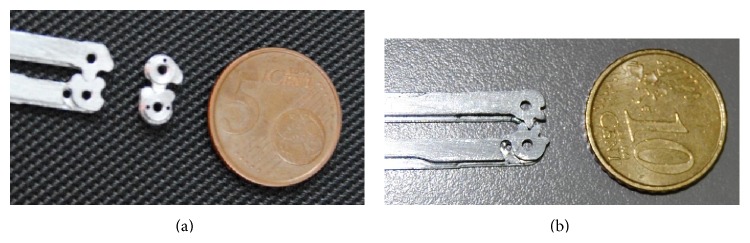
Picture of the first metal gears built.

Figure 5(a) shows the picture of the first metal pulley and gears built using a numerical milling machine, together with the picture on a coin (Euro five cents) to give an idea of the dimensions involved. In this case both the gears built fixed to the arm and the couple of gears hinged on the same shaft, to actuate the counterrotation of the frames holding forceps, are shown. These teeth had a kind of a cycloidal profile, basically impossible to build with a milling machine; thus also an involute profile was used for a second version (b), which proved to be much more reliable. Constructive parameter of the involute was thrust angle of 35°, which allowed constructing spur pinions of only eight teeth.

**Figure 6:**
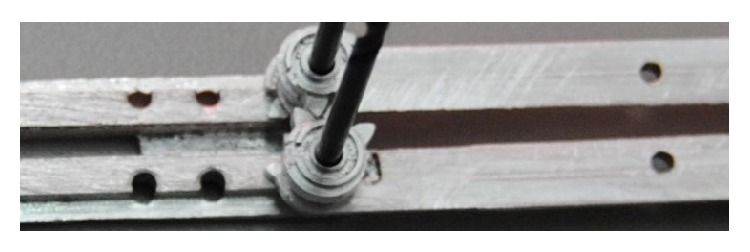
Mini gears for the system actuation.

In order to further clarify the design, Figure 6 shows a picture of the four gears mounted on the base using two drills: on the right the arms with their built-in gears, sitting on the instrument's basic support, and on the top the gears used to turn inward the frames holding forceps.

**Figure 7:**
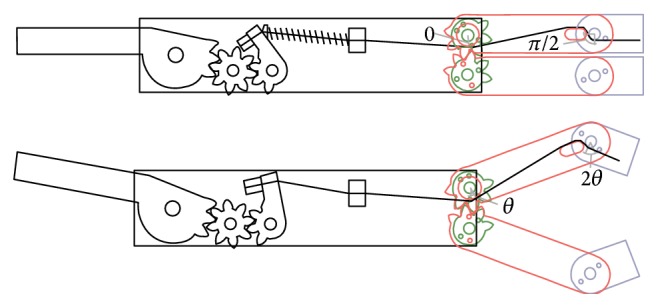
Mechanism to keep the forceps cable length constant.

Finally the two external rings just pull a cable that opens and closes the graspers. Particular attention has been devoted to the cables path, so that its length is invariant with the arms position. In fact, by introducing special pivot points, the length of the cable can still remain unaltered even when the arms can vary their degree of opening. In particular, Figure 7 shows how this can be achieved. As can be seen in fact passing from the pivot between the central body and the first arm, the cable remains always tangent to the pin of radius 2*R*, being then forced to turn by *π*/2 about a pivot of radius *R* before going to control the forceps on the auxiliary arm. Instead, when the arm is tilted, the cable should stretch by *θ* × 2*R*, while correspondingly it shortens by 2*θ* × *R* in the transition between the main and auxiliary arm, keeping its length constant.

Two were however the problems of this design. The first was the complexity of the mechanism with its tiny gears, coupled with the fact that transmitting the needed torques with these pulleys was difficult, and very often they would slip, causing misalignment of the components, which cannot be admitted in a surgical instrument. The second problem is the presence of the three actuation rings and the deriving difficulty in keeping the arms open during the surgery. This last point however can have also a positive aspect, once ratchets are used to avoid unwanted returns. In fact, using three rings and three ratchets may allow using only one hand to fully control operation of this forceps, without ever having to use a second hand. In any event, at the moment the new design uses a knob and an internal worm gear to push the arms open.

## 3. The Final Design

**Figure 8:**
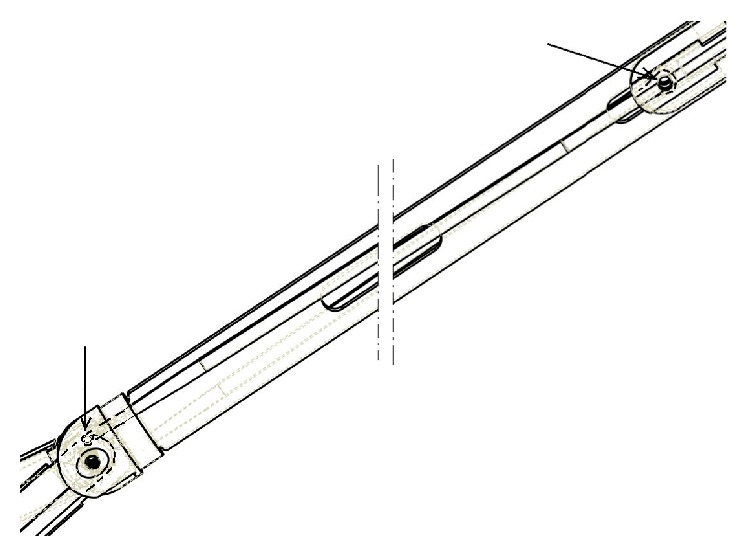
New mechanism producing arm's opening.

The solution was found in a complete redesign of the instrument, starting from the fact that the two arms, rather than being hinged on two parallel axes, are now hinged on the same axis, one above and the second below the central frame of the instrument. Moreover opening the arms was caused not any longer by cables, but by a rod pushed ahead and hinged eccentrically on the arm, as shown in Figure 8 and indicated by the arrows, where the frame has been sectioned to obtain a clearer representation [24]. Alternatively, also the model bearing the three command rings will be developed, to offer more options to the doctor, allowing single hand operation of the instrument, obviously introducing the use of ratchets and relative release button for each ring. The request of being able to move independently the arms and to orient the forceps has also been made. The first of these requests could be in principle satisfied, but at the price of introducing a fourth command, which could make this instrument a bit too complex to actuate. This might be done later on, even if it is probably not so useful, since it is always possible to vary the arms opening keeping one forceps fixed while slightly rotating the body of the instrument.

**Figure 9:**
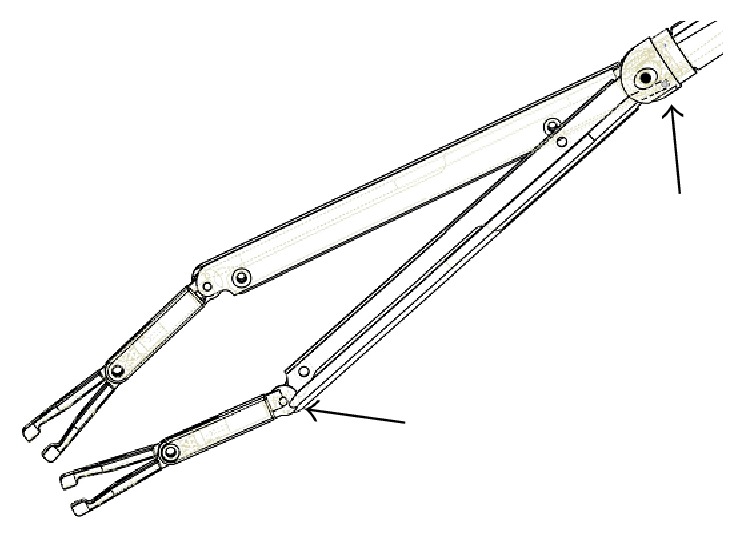
New mechanism producing forceps counterrotation.

To allow counterrotation of the mini frames holding the forceps, again a solution based on the use of a rod was found, in which the rod is hinged on a fixed point of the frame on one side and on the frame holding forceps on the other, both on the external side (Figure 9). Also in this case the two hinges are indicated in the picture by arrows, for the sake of clarity. In this way, when the arms are open, the rods push the mini frames holding the forceps that turn in the opposite direction of an angle double the rotation of the arm.

**Figure 10:**
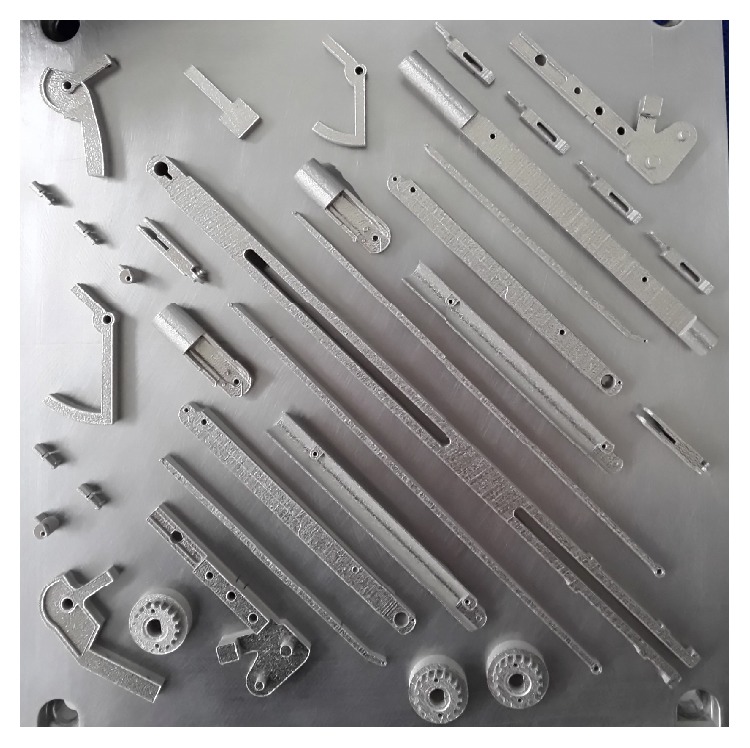
The 3D output plate holding the different components.

Given the complexity of the mechanism, all these features have been at the moment reproduced using two 3D printers by EOS, Sint M280 for steel printing, and Formiga P110 for plastic.  Figure 10 shows a plate produced in steel before detaching the components.

**Figure 11:**
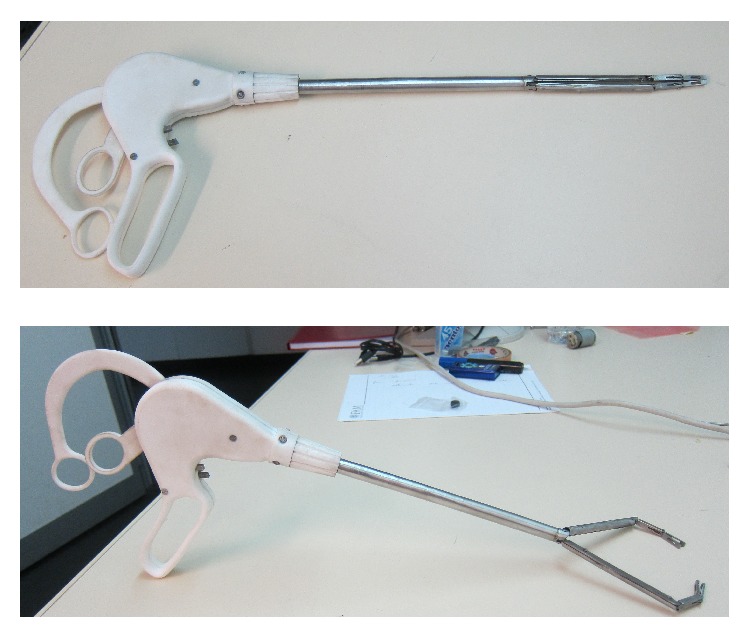
New version of the twin forceps.

Finally, in order to allow holding the arms open avoiding unwanted return, motion of the rods used to open the arms is controlled by a worm gear, reducing so the number of rings.  Figure 11 shows the last version, where the longer ring has a shape suitable not to disturb opening of the shorter one.

**Figure 12:**
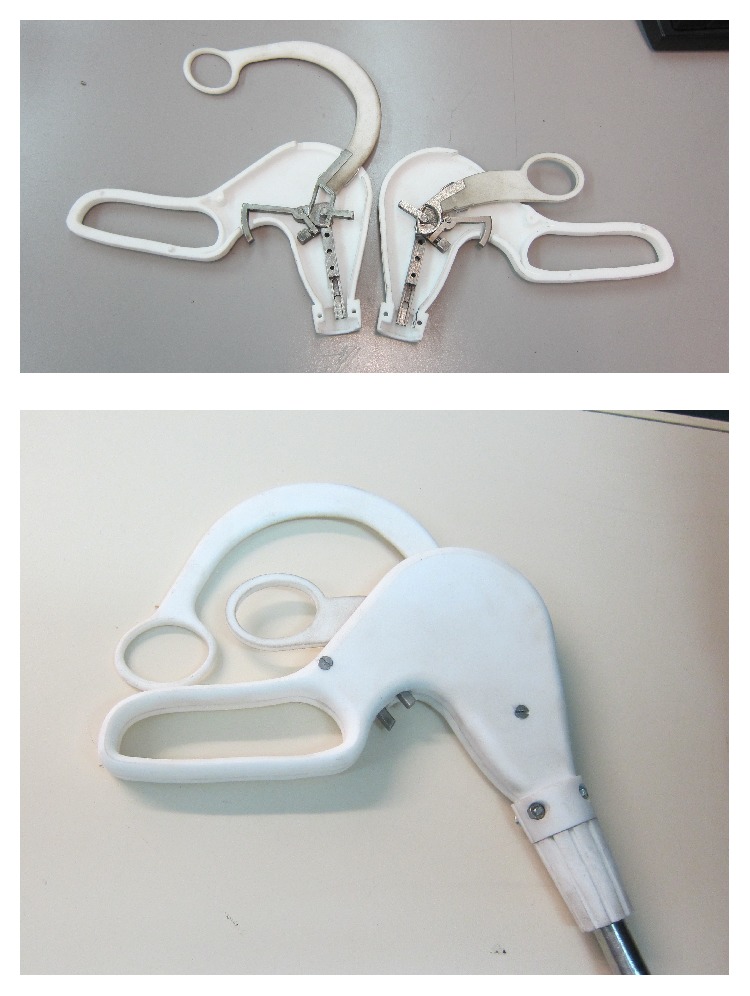
The internal ratchets and their release buttons.

This allows also the introduction of ratchet mechanism to allow locking the forceps closure and relative release buttons, shown in Figure 12.

Naturally all this has been incorporated in a new patent application [23].

Concluding this section, main shaft dimensions are 11.8 mm in external diameter and 186.6 mm in length between the end of the rotating knob and the axis of the two arms, whose dimensions are of 99.4 × 5.5 × 8.0 mm, while the graspers and relative holding frames are 47.5 × 5.5 × 5.5 mm. Only the handle, its rings, the knob, and the internal mating worm screw are plastic, everything else being made of stainless steel. Later on a disposable version will also be developed, even if some components, besides the forceps, will also be in stainless steel. Regarding pricing, this is a topic yet to be addressed.

## 4. Laboratory Training

**Figure 13:**
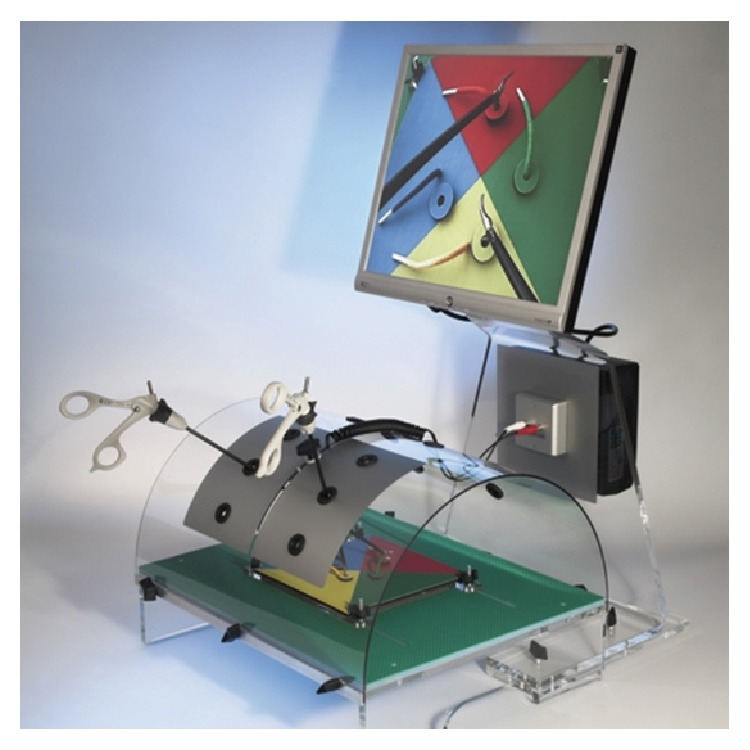
Helago laparoscopic box-trainer.

The Helago laparoscopic box-trainer is used (Figure 13). The trainer consists of laparoscopic torso with its own camera and display units with an LCD monitor. Thanks to a movable camera it is possible to change a visual angle on an open space. One side of a trainer is covered with an aluminum foil.

This arrangement allows absolute beginners control training of laparoscopic instruments under the direct sight control. In an advanced mode the control of an operation space is possible only by means of display on a screen. It can be used with a wide variety of laparoscopic instruments (5, 10, 12, and 15 mm), including graspers, thermal energy devices, and automated suturing devices [25].

The testing procedure was performed at the Surgery Department of the medical campus of Catanzaro‘s University of Magna Graecia.

### 4.1. Testing Procedure

We used the laparoscopic box-trainer to compare the innovative instrument with the classic laparoscopic instruments.

Surgeon performs two simple exercises:

1. Take the yellow string with a grasper and then insert a button with another grasper.
2. Pinch a sheet to perform a cut.

The surgeon performs these exercises first with the twin forceps and then with two classical graspers.

**Figure 14:**
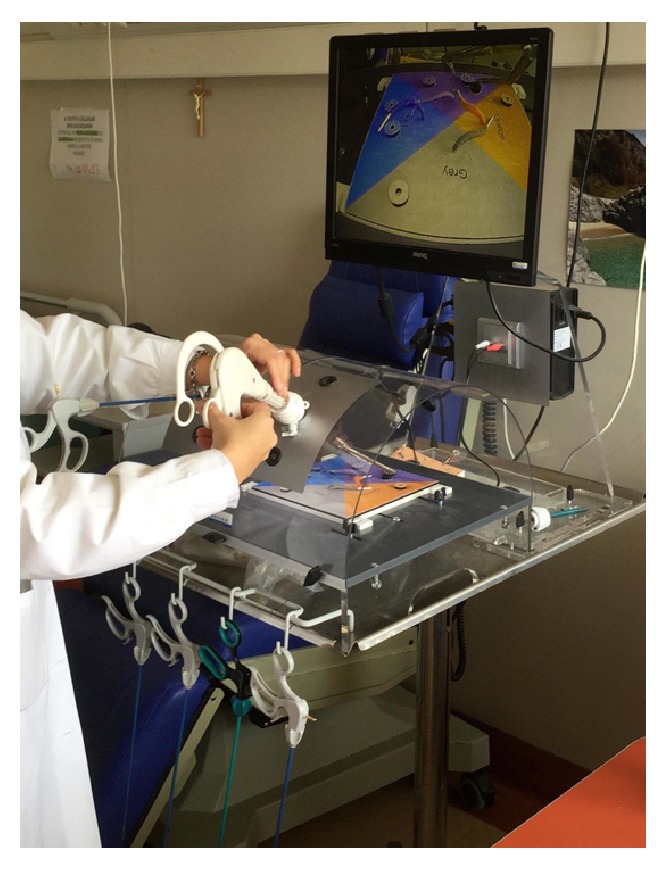
The surgeon opening the instrument's arms.

As shown in Figure 14, the surgeon inserts the double instruments into 12 mm port, following the first exercises to establish manoeuvrability of the instrument (Figure 14).

#### 4.1.1. First Exercise

**Figure 15:**
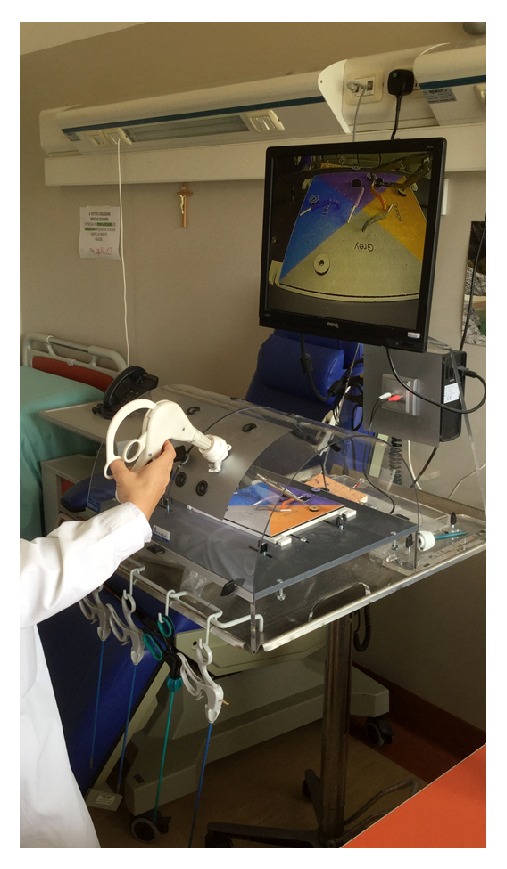
First exercise with the laparoscopic box-trainer.

**Figure 16:**
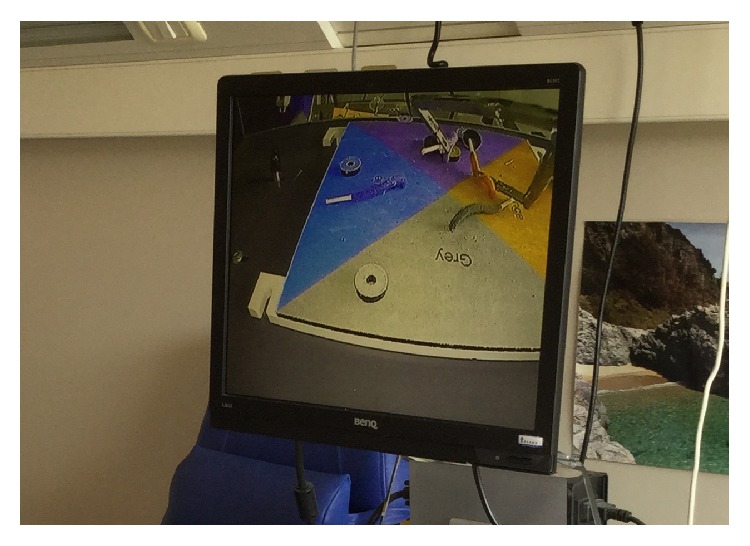
Enlargement of the screen of Figure 15; it is possible to see the two forceps holding one string each.

Surgeon inserts the double instruments into 12 mm port, following the first exercises to establish manoeuvrability of the instrument (Figures 15 and 16).

**Figure 17:**
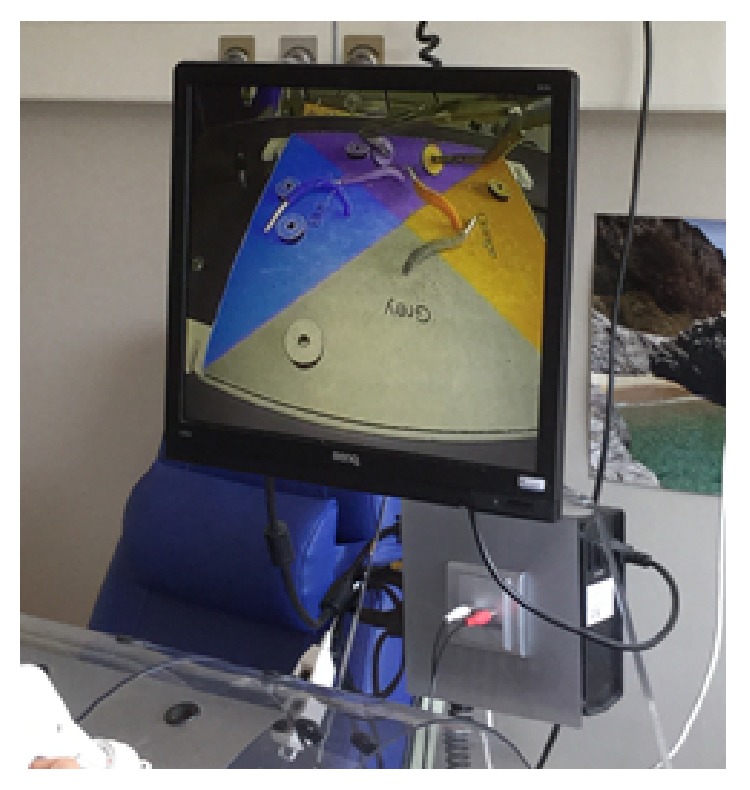
A close-up picture of the video showing the two graspers holding a button each.

To further show the possibilities of this instrument, Figure 17 shows the two forceps holding each a button.

Naturally a certain time to get acquainted to this instrument is needed; this was the first time our instrument was physically in the hand of the surgeon.

#### 4.1.2. Second Exercise

**Figure 18:**
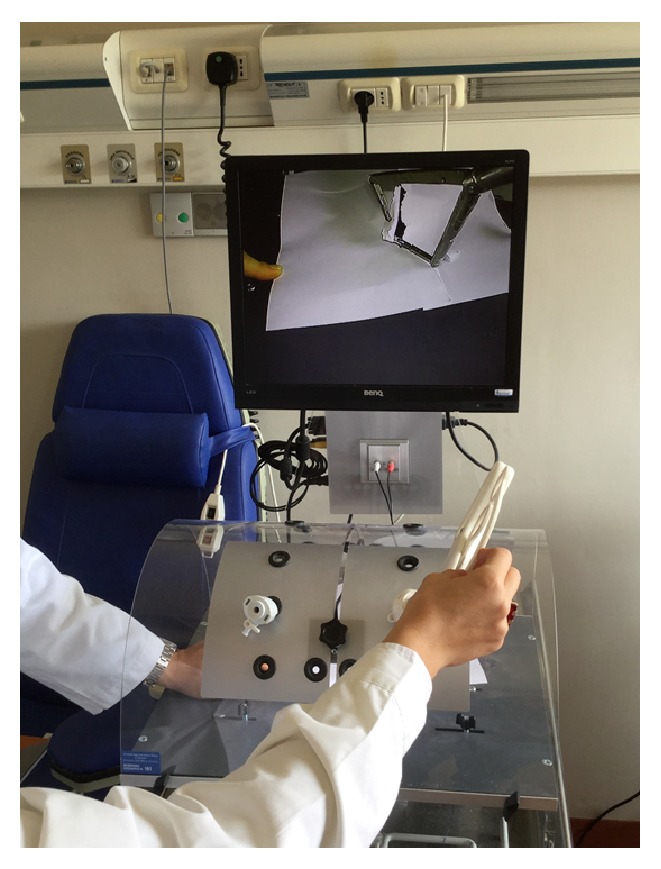
Second exercise with the laparoscopic box-trainer.

In the second exercise, the surgeon simulates the traction tissues. The surgeon uses a simple sheet in the laparoscopic box-trainer, inserts the twin forceps, and pinches two parts of this sheet.  Figure 18 shows the surgeon while performing the second exercise with a hand. Now, the surgeon can use the other hand to perform the sheet cut.

**Figure 19:**
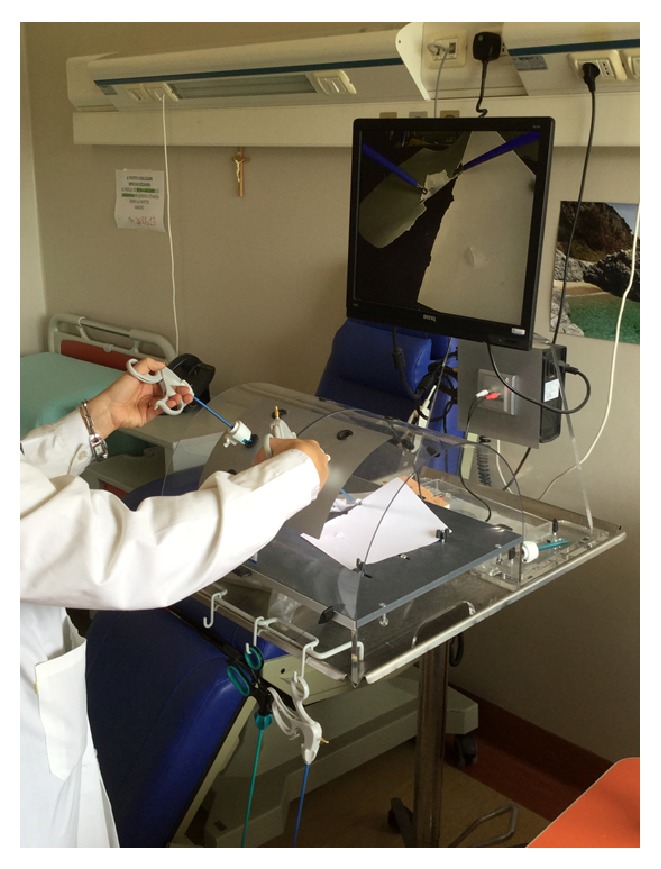
Second exercise with the laparoscopic box-trainer: the surgeon uses two instruments to perform the exercise.

Afterward, the surgeon performs the same exercise with two classical instruments.  Figure 19 shows the surgeon while performing the second exercise with two hands. In this case the surgeon has no other hand to perform a cut, so the surgeon requires another person to perform a cut.

## 5. Results

In this study, we compared the innovative instrument with the classic laparoscopic instruments. The surgeon performs two exercises using a box-trainer; for each exercise the surgeon scored the difficulty's degree using as a reference scale of 1 (easy) to 10 (difficult). The results are shown in Tables 1 and 2. It is obvious that a certain time to get acquainted to this instrument is needed; in fact it is the first time that the surgeon uses this innovative instrument.

## 6. Conclusion

This first study demonstrates that the double instruments are easy to use in SILS technique. In fact, with respect to the traditional instruments, when the surgeon uses the simulator box with the innovative graspers, the surgeon has the double tissues traction using only one port. The development of this instrument required a long time, due to the sequence of design changes, as may be observed from the PCT date, one year after the first patent application, January 2012. Due to the complexity of the design, presently we are producing the first working prototypes for technical and animal trials with a 3D printer in stainless steel, even if later on production will be mostly done with NC machines.

## Figures and Tables

**Table 1:** First exercise: insert a button.

Instrument	Size	Number of instruments	Time	Surgeon difficulty
Twin forceps	12 mm	1	13 min	5
Classic grasper	5 mm	2	5 min	1

**Table 2:** Second exercise: pinch a sheet.

Instrument	Size	Number of instruments	Time	Surgeon difficulty
Twin forceps	12 mm	1	8 min	3
Classic grasper	5 mm	2	2 min	1
